# Gene Expression Profiling of B Cell Lymphoma in Dogs Reveals Dichotomous Metabolic Signatures Distinguished by Oxidative Phosphorylation

**DOI:** 10.3389/fonc.2020.00307

**Published:** 2020-03-06

**Authors:** Ying Wu, Yu-Mei Chang, Gerry Polton, Anneliese J. Stell, Balazs Szladovits, Michael Macfarlane, Laureen M. Peters, Simon L. Priestnall, Nicholas J. Bacon, Kelvin Kow, Sarah Stewart, Eshita Sharma, Michelle R. Goulart, John Gribben, Dong Xia, Oliver A. Garden

**Affiliations:** ^1^Royal Veterinary College, London, United Kingdom; ^2^North Downs Specialist Referrals, Bletchingley, United Kingdom; ^3^Fitzpatrick Referrals, Guildford, United Kingdom; ^4^Wellcome Centre for Human Genetics, University of Oxford, Oxford, United Kingdom; ^5^Barts Cancer Institute, Queen Mary University of London, London, United Kingdom; ^6^School of Veterinary Medicine, University of Pennsylvania, Philadelphia, PA, United States

**Keywords:** diffuse large B cell lymphoma, dog, animal model, gene expression, metabolism, oxidative phosphorylation

## Abstract

Gene expression profiling has revealed molecular heterogeneity of diffuse large B cell lymphoma (DLBCL) in both humans and dogs. Two DLBCL subtypes based on cell of origin are generally recognized, germinal center B (GCB)-like and activated B cell (ABC)-like. A pilot study to characterize the transcriptomic phenotype of 11 dogs with multicentric BCL yielded two molecular subtypes distinguished on the basis of genes important in oxidative phosphorylation. We propose a metabolic classification of canine BCL that transcends cell of origin and shows parallels to a similar molecular phenotype in human DLBCL. We thus confirm the validity of this classification scheme across widely divergent mammalian taxa and add to the growing body of literature suggesting cellular and molecular similarities between human and canine non-Hodgkin lymphoma. Our data support a One Health approach to the study of DLBCL, including the advancement of novel therapies of relevance to both canine and human health.

## Introduction

Diffuse large B cell lymphoma (DLBCL), an aggressive malignancy of mature B lymphocytes, comprises the most common subtype of non-Hodgkin lymphoma (NHL) (1). Variable clinical characteristics and treatment response of patients with DLBCL have prompted investigations into its molecular heterogeneity (1–3). Gene expression profiling has classified DLBCL into multiple subtypes: the cell-of-origin (COO) classification identifies three subsets named germinal center B (GCB)-like, activated B cell (ABC)-like, and “undefined,” while the consensus cluster classification (CCC) identifies three subsets named “oxidative phosphorylation (OxPhos),” “B cell receptor/proliferation,” and “host response” (2, 3). The COO and CCC classification schemes yield complementary information and differentially stratify patients, with little correlation or overlap (3, 4).

Dogs have been increasingly gaining traction as an animal model in comparative oncology, attributed to their spontaneous development of cancer, intact immune system, and shared living environment with humans, with common exposure to xenobiotics (5). Canine DLBCL mirrors human DLBCL in clinical presentation, therapeutic modalities, and molecular pathogenesis (6, 7). A number of studies have set out to dissect the molecular signature of canine DLBCL, aiming to better diagnose this cancer and predict treatment outcome for patients of both species. For instance, our previous study showed that overexpression of FoxP3 by intratumoral T cells in canine multicentric BCL correlates with shorter survival (8). A gene profiling study has revealed GCB-like and ABC-like subtypes in canine DLBCL, the latter of which shows less favorable survival, thus resembling the human disease (9). The same study also proposed a list of 1,180 genes to distinguish GCB-DLBCL and ABC-DLBCL in dogs, prompting us to question whether a shorter list of selective classifiers may serve the same purpose. We therefore undertook a pilot study to analyze the transcriptomic phenotype of dogs with multicentric BCL, all with the cytological characteristics of DLBCL, hypothesizing that molecular subtype may be identified by a selective subset of gene identifiers.

## Materials and Methods

### Sample Collection

Samples of 11 canine lymphoma cases (Supplementary Data 1) were collected in a sterile fashion by qualified veterinarians prior to chemotherapy, with signed informed consent of the owner and approval of the Royal Veterinary College (RVC) Ethics and Welfare Committee (Permit Number: URN 2014 1285) in the United Kingdom (UK). Dogs of any age, breed, gender, or neutering status were recruited to minimize selection bias. Cells aspirated from enlarged peripheral lymph nodes were flushed into 100% fetal bovine serum (Biosera, East Sussex, UK) for immunophenotyping by flow cytometry. Board-certified clinical pathologists undertook contemporaneous cytological review. All 11 cases were confirmed to be BCL, with cytological features of DLBCL.

### Cell Isolation

Aspirated lymph node cells were labeled by a mixture of R-phycoerythrin (PE)-conjugated anti-dog CD5 (clone YKIX322.3; Bio-Rad, UK), Alexa Fluor 647®-conjugated anti-dog CD21 (clone CA2.1D6; Bio-Rad) and 4′,6-diamidino-2-phenylindole (DAPI; BioLegend, San Diego, CA, USA). CD5^−^CD21^+^ cells, the majority of which comprised neoplastic B cells, were isolated using fluorescence-activated cell sorting (FACS™). RNA extraction, sequencing, and read processing of the isolated B cells were performed in accordance with our previous protocols (10).

### RNA Extraction and Sequencing

Total RNA was extracted from FACS™-isolated CD5^−^CD21^+^ cells using RNA Bee (AMS Biotechnology, Abingdon, UK) and Direct-zol™ RNA MicroPrep Kit (Zymo Research, Irvine, CA, USA), according to the manufacturer's instructions. All samples were treated with DNase I during extraction and examined by Agilent 2100 Bioanalyzer (Agilent Technologies, Santa Clara, CA, USA), with an RNA Integrity Number of greater than or equal to 6.5. Seventy-five-bp, paired-end RNA sequencing (RNA-seq) was performed using the HiSeq.4000 System (Illumina, San Diego, CA, USA) at the Oxford Genomics Centre, University of Oxford (Oxford, UK). RNA-seq read processing and expression quantification followed methods used in our previous study (10). Quality control was performed by the Oxford Genomics Centre to assess sample processing and data integrity. Transcript reads were mapped and annotated to the canine genome, CanFam3.1 (Ensembl Genes, release 91). Read counts were all normalized to transcripts per million (TPM) values for subsequent data analyses.

### Data Analyses

Hierarchical cluster analysis was conducted on *Morpheus* (Broad Institute, USA) using the one minus Pearson correlation and average linkage method. Principal component analysis (PCA), volcano plot creation, and survival analysis were all undertaken in R (version 3.4.2; R code provided in Supplementary Data 2). Differential expression analysis was performed using the Bioconductor package edgeR (Bioconductor version 3.6) (11). Differentially expressed genes with a false discovery rate (FDR) of <0.05 were input into the software Ingenuity Pathway Analysis (IPA; Ingenuity Systems Inc., Redwood City, CA, USA) to identify biological pathways affected by the altered expression of these genes (*p* < 0.05, |Z| score ≥ 2). Overall survival time post-diagnosis was estimated for nine of the 11 dogs using Kaplan-Meier curves and Cox regression analysis in R. (Dogs 7 and 11 received no chemotherapy for BCL and were excluded from survival analysis.)

### Comparison With Human Dataset

Processed microarray data of 203 human DLBCL samples analyzed by Affymetrix Human Genome U133 Plus 2.0 Array (GEO accession number GSE11318) were downloaded (12). To combine the 11 canine RNA-seq and 203 human microarray data, a list of 14,224 consensus genes were identified between the two datasets based on annotated gene symbols. Canine RNA-seq data were z-transformed, and human microarray data were log_2_-transformed followed by z-transformation. Normalized expression data of 22 genes were retrieved from the combined datasets on the basis of the previously identified 27-gene classifiers that distinguish human GCB- and ABC-DLBCL (2, 9), and analyzed on *Morpheus* using K-means (K = 2) and average linkage hierarchical clustering.

## Results

### Gene Expression Profiling Reveals Two Molecular Subtypes of Canine B Cell Lymphoma With Distinct Metabolic Signatures

**Figure 1:**
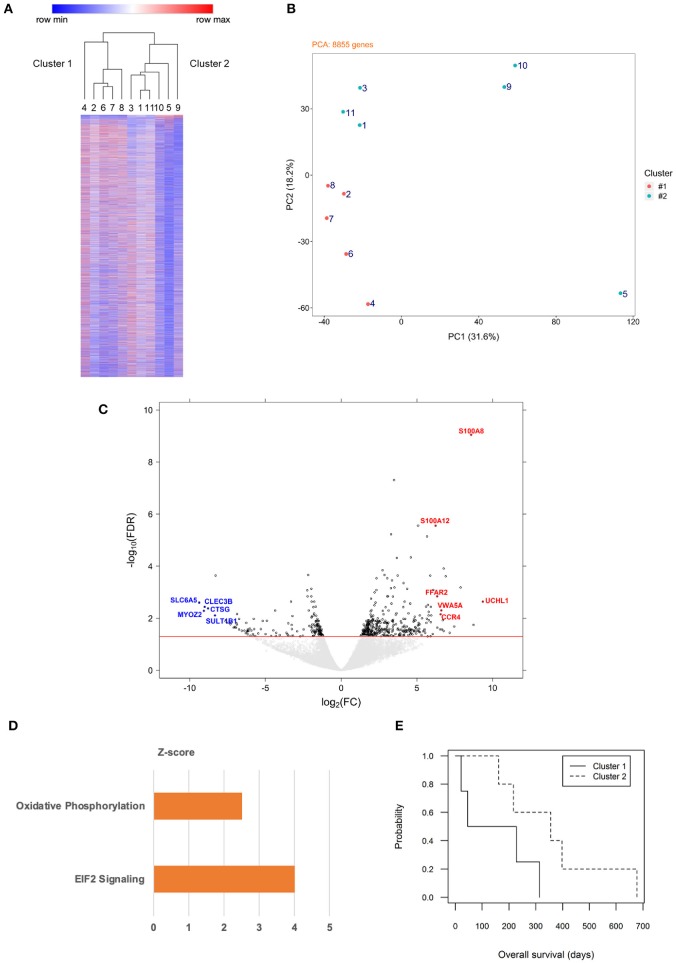
Gene expression profiling of canine B cell lymphoma with distinct metabolic signatures distinguished by oxidative phosphorylation. **(A)** Unsupervised hierarchical clustering classifies genome-wide expression data of 11 dogs with multicentric BCL into two major clusters. Each row represents one gene, color intensity positively correlating with TPM values presented by rows in relative value from 0 to 1. Numbers 1–11 represent each of the dogs. The two major clusters are designated 1 (left) and 2 (right). **(B)** Principal component analysis of genome-wide expression data of 8,855 genes from the same 11 dogs confirms spatial segregation of the two major clusters (cluster 1: orange; cluster 2: teal). **(C)** A volcano plot of expression data of differentially expressed genes of dogs in cluster 2 (*n* = 6) vs. dogs in cluster 1 (*n* = 5) identifies genes of particular interest. The threshold line (red) indicates a false detection rate of 0.05; each dot represents one gene (to facilitate visualization, gene symbols are designated in upper case). **(D)** Ingenuity Pathway Analysis of differentially expressed genes (cluster 2 vs. cluster 1) reveals significant enrichment of two biological pathways (*p* < 0.05), one focusing on oxidative phosphorylation (orange color represents activated status). **(E)** Overall survival time post-diagnosis was compared between clusters 1 and 2 using Kaplan-Meier curves and Cox regression analysis (*p* = 0.12).

Unsupervised hierarchical clustering using genome-wide expression data of the neoplastic B cells segregated the 11 cases into two major clusters (Figure 1A). Their distinct expression signatures were confirmed by PCA (Figure 1B). We then set out to analyze genes differentially expressed between the two clusters and identified a list of 527 genes (FDR <0.05), of which 162 were of lower transcript abundance and 365 were of higher transcript abundance in cluster 2 compared to cluster 1 (Figure 1C). One hundred and forty-six (~28%) of the 527 genes are involved in mitochondrial metabolism or function, of which 50 were of lower transcript abundance and 96 were of higher transcript abundance in cluster 2 vs. cluster 1 (Supplementary Data 3). To identify genes with interesting functional implications, we narrowed down this list of 527 genes on the basis of two criteria. We first identified genes differentially expressed with high significance (FDR <0.01). We then identified genes in this curated list that were most variably expressed, i.e., in the top or bottom third of the list. As a result, five genes of lower transcript abundance and six genes of higher transcript abundance in cluster 2 were selected (Figure 1C). Of the 11 selected genes, *uchl1, s100a8*, and *s100a12* are of particular interest, all correlating with oxidative stress (13, 14). Seven of the remaining eight genes have been associated with various cancer contexts, consonant with their association with subsets of canine DLBCL. Hence, *ccr4* (15, 16), *myoz2* (17), and *clec3b* (18, 19) are adverse prognosis biomarkers, while *vwa5a* (20) and *sult1b1* (21) serve a tumor suppressor role in human solid tumors. The gene *ffar2* encodes a short chain fatty acid receptor that may inhibit metastasis of human breast cancer cells (22), and cathepsin-G encoded by *ctsg* is a neutrophil protease that facilitates cell adhesion in human breast cancer cells (23). The remaining gene *slc6a5* encodes a glycine transporter protein whose loss of function is implicated in human hyperekplexia (24, 25).

Ingenuity Pathway Analysis of the 527 differentially expressed genes revealed that both OxPhos and eukaryotic initiation factor (EIF) 2 signaling pathways were enriched and activated in cluster 2 (Figure 1D). Overall survival time post-diagnosis was analyzed: dogs from cluster 1 had an estimated median survival time of 137 days, while those from cluster 2 had a median survival time of 356 days, with a hazard ratio of 0.26 (cluster 2 vs. cluster 1; 95% confidence interval: 0.05–1.44; *p* = 0.12; Figure 1E). Furthermore, among the 131 genes functionally annotated in “OxPhos” in the human database (Systematic name: M19540, Gene Set Enrichment Analysis, Broad Institute), 80 genes were annotated in the canine genome (CanFam3.1), of which six were of higher transcript abundance and four were of lower transcript abundance in canine cluster 2 vs. cluster 1 (Supplementary Data 4). These results collectively suggest that the 11 dogs with multicentric BCL in our study fell into two molecular subgroups with distinct metabolic signatures characterized by OxPhos, with no significant difference in survival. We therefore designated cluster 1 as “non-OxPhos” and cluster 2 as “OxPhos” subgroups in the remainder of this study.

### *Cell of origin* and *Metabolic* Molecular Classification Schemes of B Cell Lymphoma in Dogs Differentially Segregate Cases

**Figure 2:**
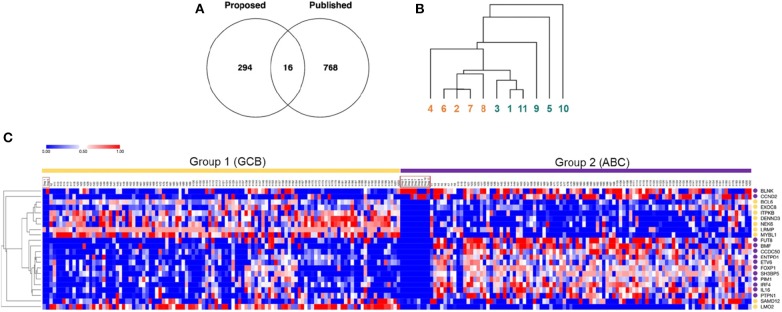
*Cell of Origin* and *Metabolic* molecular classification schemes of B cell lymphoma in dogs differentially segregate cases. **(A)** A Venn diagram of common and distinct differentially expressed genes between our dataset (*n* = 527 genes) and the published canine GCB/ABC-DLBCL gene classifier dataset (*n* = 1,180 genes) reveals minimal overlap of the two classification methods. **(B)** Hierarchical cluster analysis of B cell gene expression data from dogs recruited in this study using the published 1,180 canine GCB/ABC-DLBCL gene classifiers fails to stratify the cases into two major subtypes, suggesting a lack of distinction on the basis of cell of origin (numbers 1–11 represent each of the dogs. Colors indicate the original cluster of the 11 dogs, as identified in Figure 1. Redundant and un-annotated genes were excluded from all analyses). **(C)** Co-clustering of B cell gene expression data from 11 canine RNA-seq data in this study and published human microarray data (*n* = 203). The heatmap was generated *via* clustering combined datasets by columns using K-means (K = 2) and by rows using average linkage hierarchical clustering. Each column represents one sample, and each row represents one gene (color intensity positively correlating with z-transformed expression values presented by value from 0 to 1; gene symbols are designated in upper case to facilitate visualization). Sample names are annotated on top of the heatmap, with the 11 canine samples highlighted by red boxes. Dogs 1 and 5 co-clustered with 106 human samples falling into group 1, whereas the remaining nine dogs co-clustered with 97 human samples falling into group 2. Yellow circles annotate subtype-classifying genes highly expressed in human GCB-DLBCL, and purple circles annotate genes highly expressed in human ABC-DLBCL.

We then compared our 527 genes with the published 1,180 canine DLBCL COO subtype-classifiers, following the exclusion of redundant and un-annotated data (Figure 2A). The paucity of consensus genes, numbering only 16, suggested limited overlap between the two lists (Supplementary Data 5). Unsupervised hierarchical clustering using the published 1,180 classifiers failed to segregate our transcriptomic data into two major clusters (Figure 2B), further confirming the lack of consensus and inability of the COO classification scheme to stratify our cases. We speculated that this could have reflected the limited number of cases, with a majority bias toward one COO subtype or the other. Therefore, we co-clustered our 11 canine RNA-seq and 203 human DLBCL microarray data on the basis of the previously identified 27-gene classifiers that distinguish human GCB- and ABC-DLBCL (2, 9). Five of the 27 genes were excluded from this analysis: *tbc1d27* had no canine homolog; *c1orf186, mme*, and *serpina9* were expressed in none or fewer than two of the 11 dogs; and *ighm* had a predominant expression in some of the dogs that biased the clustering (9). K-means (K = 2) clustering partitioned the combined 11 canine and 203 human samples into two groups: two dogs co-clustered with 106 human samples in group 1, and the other nine dogs co-clustered with remaining 97 human samples in group 2 (Figure 2C). Hierarchical clustering on the basis of the 22 human GCB/ABC classifier genes revealed distinct expression patterns of the two groups: genes highly expressed in GCB-DLBCL were enriched in group 1, while genes highly expressed in ABC-DLBCL were enriched in group 2 (Figure 2C). Taken together, these results suggested that the two dogs in group 1 were more likely to be of GCB phenotype, while the remaining nine dogs in group 2 were more likely to be of ABC phenotype, consistent with a skewed COO phenotypic distribution of the canine patients.

## Discussion

This pilot study set out to characterize the transcriptomic signature of 11 dogs with DLBCL, aiming to refine the previously published canine DLBCL-subtyping COO classifier list. Our results suggest that multicentric BCL in dogs may be stratified in an alternative, non-overlapping manner based on metabolic signatures rather than COO characteristics. One of the two identified subtypes resembles the “OxPhos”-DLBCL subtype in human patients (4). We therefore propose a novel, complementary molecular classification of canine BCL, named OxPhos and non-OxPhos, with an understanding of several caveats to be addressed by future work. First, the small sample size of cases in our study may not adequately represent the diversity of canine DLBCL. Distinction of the two subtypes needs prospective validation in a larger cohort of cases using the 527 differentially expressed genes. Second, genes involved in oxidative metabolism are not all enriched in the proposed canine OxPhos subtype, as they are in the human counterpart. Third, the COO classification scheme has been refined in human DLBCL: four gene expression signatures, termed as “germinal center B cell,” “proliferation,” “major histocompatibility complex (MHC) class II,” and “lymph node” correlate with prognosis of patients treated by CHOP chemotherapy (26). The parallelism of these molecular subtypes in the two species therefore needs further, rigorous investigation. Survival times between the two subgroups showed no significant difference in this study, although the small sample size introduced the possibility of a type II error. Moreover, several dogs received non-CHOP chemotherapy, further confounding analysis of survival. Correlation of metabolic signature with prognosis is still unclear in human DLBCL: one study showed similar five-year survival between patients with OxPhos- and non-OxPhos-DLBCL (3), whereas another revealed poor response to rituximab (R)-CHOP treatment in human OxPhos-DLBCL (27). The prognosis of the OxPhos signature in canine DLBCL, and its resemblance to the human counterpart, therefore needs further characterization in a larger and more homogeneous cohort of cases.

Neoplastic cells reprogram their metabolism to sustain high proliferation (28, 29) and to resist apoptosis induced by oxidative stress (30). Numerous studies have been conducted to investigate the metabolic reprogramming of neoplastic cells, aiming to identify potential therapeutic targets (31). Major ATP production shifts from mitochondrial respiration to aerobic glycolysis in a number of tumors, a process termed the Warburg phenomenon (32, 33). However, some tumors rely on oxidative metabolism as their main energy source in certain contexts (34), including melanomas (35), lymphomas (36), and pancreatic (37) and pulmonary carcinomas (38). A variety of common Food and Drug Administration-approved drugs, such as metformin, arsenic trioxide, and atovaquone, act as OxPhos inhibitors and have potential as anti-neoplastic drugs, especially in the setting of tumors with unregulated oxidative phosphorylation (39). Moreover, mammalian target of rapamycin (mTOR) is a pivotal serine/threonine kinase upstream of metabolic pathways, including OxPhos, and thus also constitutes a novel therapeutic target (40, 41). One of its complex forms, mTORC1, is deregulated in DLBCL (42). mTORC1 inhibitors, known as rapalogs, kill DLBCL cell lines *in vitro*, but unfortunately show limited efficacy in patients with DLBCL, underlining the continuing unmet need for novel treatments targeting this molecular pathway (42). The three genes *uchl1, s100a8*, and *s100a12*, enriched in the canine OxPhos subgroup of our study, have interesting functional implications. *Uchl1* encodes ubiquitin C-terminal hydrolase L1 (UCHL1), a de-ubiquinating enzyme that plays a pivotal role in maintaining cellular homeostasis under physiological conditions of oxidative stress (13). UCHL1 has variable roles in cancers, depending on histotype: for instance, the expression of UCHL1 correlates with poor prognosis in patients with multiple myeloma (43), mammary carcinoma (44), and pulmonary carcinoma (44), but has tumor suppressive properties in nasopharyngeal (45) and prostatic carcinoma (46). Both S100A8 and S100A12 are members of the calcium-binding S100 protein family (47), expression of which correlates with inflammation-associated carcinogenesis (48). However, S100A8 may also be anti-inflammatory and acts as an oxidant scavenger during oxidative stress and inflammation (14). Moreover, high expression of S100A8 and S100A12 has a positive prognostic impact on oropharyngeal squamous cell carcinoma (48). The functions of S100A8 and S100A12 are therefore broad and context-dependent. Taken together, these three genes may be potential biomarkers and therapeutic targets of OxPhos-DLBCL in both dogs and humans, given their prognostic impact in other cancers and correlation with oxidative stress imposed by an abundance of reactive oxygen species as a consequence of vigorous OxPhos (49). Furthermore, EIF2 signaling is induced by various cellular stresses, including those of oxidative origin, consolidating the view that this molecular subtype is distinguished by overactive OxPhos.

Future work will aim to provide additional insight into the genes *uchl1, s100a8*, and *s100a12* in both canine and human DLBCL to confirm observations made in the current pilot study. First, distinction of OxPhos- and non-OxPhos-DLBCL will be validated in a larger cohort of canine cases using the 527 differentially expressed genes. Second, protein expression of the three genes will be examined in OxPhos- vs. non-OxPhos-DLBCL in both species, using immunohistochemistry in conjunction with flow cytometry and Western blots. The genes with a conserved expression pattern may be investigated for their functional impact in knockdown studies performed *in vitro* and *in vivo*, the latter requiring rodent models. Third, expression of the three genes may also be examined in DLBCL subtypes identified by the COO scheme to further interrogate the degree of overlap and prognostic significance of these two classification schemes. For instance, high expression of *uchl1* identifies human GCB-DLBCL patients likely to have a poor outcome (50). Meticulous longitudinal studies in both species will ultimately yield a better understanding of the prognostic impact of *uchl1, s100a8*, and *s100a12* in DLBCL.

In summary, our study has revealed hitherto unrecognized metabolic heterogeneity of multicentric BCL in dogs that resembles that of human DLBCL. These data yield potentially interesting therapeutic targets for canine lymphoma and substantiate the dog as a model for human NHL, further validating its use in the advancement of novel therapies of relevance to human health.

## Data Availability Statement

Raw and processed RNA-seq data of the 11 canine samples in this study have been deposited to Gene Expression Omnibus (GEO), accession number GSE140603.

## Ethics Statement

The animal study was reviewed and approved by Royal Veterinary College (RVC) Ethics and Welfare Committee (Permit Number: URN 2014 1285) in the United Kingdom. Written informed consent was obtained from the owners for the participation of their animals in this study.

## Author Contributions

YW conducted the entire study and wrote the initial draft of the manuscript. Y-MC performed survival analysis and volcano plot gene annotation. GP, AS, NB, KK, and MM recruited and sampled clinical cases. BS and LP conducted cytological review of all clinical cases and making the definitive diagnoses. AS and SP co-supervised YW on project conduct and data interpretation. SS followed up outcome of canine patients at the RVC. ES performed initial RNA-seq analysis and provided scripts for basic transcriptomic analysis. MG helped with initial outcome data input in survival analysis. JG co-supervised YW and co-funded the study. DX contributed expertise and intellectual input on all the transcriptomic data interpretation, and co-supervised YW in the last year of the study. OG conceived and funded the entire study, served as the principal supervisor of YW, and edited the initial manuscript. All authors reviewed and approved the final manuscript.

### Conflict of Interest

The authors declare that the research was conducted in the absence of any commercial or financial relationships that could be construed as a potential conflict of interest.
