# Supplementary material for: Gene Expression Profiling of B Cell Lymphoma in Dogs Reveals Dichotomous Metabolic Signatures Distinguished by Oxidative Phosphorylation
Source: Front Oncol. 2020 Mar 6;10:307. doi: 10.3389/fonc.2020.00307 (PMC7069556; doi:10.3389/fonc.2020.00307)
Supplement: Supplementary file 1 [file Table_1.DOCX]

**Supplementary Data 1: Eleven dogs with B cell lymphoma are recruited to this study**

| **Dog No.** | **Age** | **Breed** | **Sex** | **Neuter Status** | **Chemotherapy** |
| --- | --- | --- | --- | --- | --- |
| 1 | 4 y^1^ | Irish Water Spaniel | F^3^ | N^5^ | CHOP^7^ |
| 2 | 7 y 11 mo^2^ | Mastiff | M^4^ | E^6^ | CHOP |
| 3 | 5 y 11 mo | Dachshund Crossed | M | E | CHOP |
| 4 | 5 y | Staffordshire Bull Terrier | M | N | CHOP |
| 5 | 1y 10 mo | Border Collie | M | N | CHOP |
| 6 | 5 y | Dobermann | M | N | CHOP |
| 7 | 12 y | Cross Breed | F | N | N/A^8^ |
| 8 | 7 y | Cross Breed | F | N | Non-CHOP^9^ |
| 9 | 5 y | Golden Retriever | M | N | COP^10^ |
| 10 | 6 y | Cocker Spaniel | F | N | CHOP |
| 11 | 10 y | Beagle | F | N | N/A |

Abbreviations: ^1^Year; ^2^Month; ^3^Female; ^4^Male; ^5^Neutered; ^6^Entire; ^7^Cyclophosphamide, doxorubicin, vincristine and prednisone/prednisolone; ^8^Not available (untreated); ^9^L-asparaginase, chlorambucil and prednisolone; ^10^cyclophosphamide, vincristine and prednisone/prednisolone
