# Supplementary material for: Gene Expression Profiling of B Cell Lymphoma in Dogs Reveals Dichotomous Metabolic Signatures Distinguished by Oxidative Phosphorylation
Source: Front Oncol. 2020 Mar 6;10:307. doi: 10.3389/fonc.2020.00307 (PMC7069556; doi:10.3389/fonc.2020.00307)
Supplement: Supplementary file 2 [file Table_2.DOCX]

**Supplementary Data 2: R Code for Principal Component Analysis, Volcano Plot and Survival Analysis.**

#########################################

#### 1. Principal component analysis ##########

#########################################

library(ggplot2)

library(ggrepel)

library(reshape2)

library(grid)

theme_QC <- theme(panel.background = element_rect(fill = "white", colour = NA),

panel.grid.major = element_line(linetype = 'blank'),

panel.grid.minor = element_line(linetype = 'blank'),

axis.text.x=element_text(colour="black",size=14),

axis.text.y=element_text(colour="black",size=14),

axis.title.x = element_text(vjust=-2),

axis.title.y = element_text(vjust=2),

legend.text=element_text(size=14),

plot.title = element_text(size=14,vjust=2,colour="darkorange1"),

strip.text.x = element_text(size = 14),

plot.margin=unit(c(1,0,0.5,0.5),'cm'))

calc.pca <- function(data)

{

pca.norm <- prcomp(t(data))

PC.norm <- pca.norm$x

PC.norm <- data.frame(PC.norm)

attach(PC.norm)

var.cap <- summary(pca.norm)

pc_var.cap <- round(var.cap$importance[2,]*100, 1)

cum_var.cap <- round(var.cap$importance[3,]*100, 1)

out <- list("PC"=PC.norm, "PC1_var"=pc_var.cap[1], "PC2_var"=pc_var.cap[2], "PC_var"=pc_var.cap,"Cum_var"=cum_var.cap)

return(out)

}

tpm.expressed <- function (TPM,filter=filter,num.samples=num.samples,

...)

{

keep <- rowSums(TPM >= filter) >= num.samples

TPM <- TPM[keep,]

return(TPM)

}

#set working drive. Change it to your local directories.

setwd("XXXX")

#read results table into R.

tpm <- read.table("tpm_gene_count_table.txt",sep=" ",header=T)

layout <- read.delim("cell_annotation.txt")

factor_columns <- c("sampleID","Project.no.","Patient.names","Disease","Cluster","Cell.type")

layout$Library <- as.character(layout$Library)

tpm_exp <- tpm.expressed(tpm,1,10)

genes <- (dim(tpm_exp))[1]

pc.fil <- calc.pca(log2(tpm_exp+1))

plot_data <- pc.fil$PC

plot_data[,factor_columns] <- layout[match(rownames(plot_data),layout$Library),factor_columns]

pca_plot <- ggplot(plot_data,aes(y=(PC2),x=(PC1)))+

geom_point(aes(colour=Cluster),size = 2.5) +

geom_text_repel(aes(label=sampleID), size=5.7,col="navy", nudge_x=0.05)+

theme_QC +

theme(panel.background=element_rect(colour="black"))+

theme(text = element_text(size=16))+

labs(x=paste0("PC1 (",pc.fil$PC1_var,"%)"),

y=paste0("PC2 (",pc.fil$PC2_var,"%)"))+

scale_shape_manual(values=c(17,16,15,18,19))+

ggtitle(paste0("PCA: ",genes," genes"))

width <- 25

height <- 20

tiff(filename = "pca_plot.tif",

width = width, height = height, unit="cm", compression = "lzw", bg = "white", res = 600)

print(pca_plot)

dev.off()

#########################

#### 2. Volcano plot ########

#########################

library(lattice)

library(latticeExtra)

#set working drive. Change it to your local directories.

setwd("XXXX")

#read results table into R.

edgeR.res <- read.table("EdgeR_result.txt",sep=" ",header=T)

#separate results table into up and down using subset.

data.up <- droplevels(subset(read.table("../EdgeR_result_Up_genes.txt",

sep="\t",header=T),Plot==1, drop=T))

colnames(data.up)[1] <- "genes"

data.down <- droplevels(subset(read.table("../EdgeR_result_Down_genes.txt",

sep="\t",header=T),Plot==1, drop=T))

colnames(data.down)[1] <- "genes"

data.up <- subset(data.up, Symbol %in% c("UCHL1", "S100A8", "VWA5A", "CCR4", "FFAR2", "S100A12"))

data.down <- subset(data.down, Symbol %in% c("SLC6A5", "MYOZ2", "CLEC3B", "CTSG", "SULT1B1"))

data.up$logFDR <- -log10(data.up$FDR)

data.down$logFDR <- -log10(data.down$FDR)

#Annotated 6 Up genes: UCHL1, S100A8, VWA5A, CCR4, FFAR2, S100A12 (in red)

#Annotated 5 Down genes: SLC6A5, MYOZ2, CLEC3B, CTSG, SULT1B1 (in blue)

fig.volcano <- xyplot(-log10(FDR) ~ logFC, data=edgeR.res,xlim=c(-12,12), ylim=c(-0.5,10.5),

xlab=list(cex=1.2,label=expression(paste("log"[2],"(FC)"))),

ylab=list(cex=1.2,label=expression(paste("-log"[10],"(FDR)"))),

scale=list(cex=1.1),

pch=1,cex=0.5,col="black",subset=(FDR<0.05))+

xyplot(-log10(FDR) ~ logFC, data=edgeR.res,xlim=c(-12,12), ylim=c(-0.5,10.5),

pch=1,cex=0.5,col="grey90", subset=(FDR>=0.05),

panel=function(x,y,...){

panel.xyplot(x,y,...)

panel.abline(h=(1.30),lty=1,col="red")})+

xyplot(-log10(FDR) ~ logFC, data=data.up,xlim=c(-12,12), ylim=c(-0.5,10.5),

pch=16,cex=0.5,col="red",

panel=function(...){

panel.xyplot(...)

panel.text(x=10.05, y=2.68,label="UCHL1",col="red",cex=0.7,fontface="bold")

panel.text(x=8.65, y=9.25,label="S100A8",col="red",cex=0.7,fontface="bold")

panel.text(x=6.75, y=2.5,label="VWA5A",col="red",cex=0.7,fontface="bold")

panel.text(x=7.05, y=2.05,label="CCR4",col="red",cex=0.7,fontface="bold")

panel.text(x=6.3, y=3.05,label="FFAR2",col="red",cex=0.7,fontface="bold")

panel.text(x=6.2, y=5.8,label="S100A12",col="red",cex=0.7,fontface="bold")

})+

xyplot(-log10(FDR) ~ logFC, data=data.down,xlim=c(-12,12), ylim=c(-0.5,10.5),

pch=16,cex=0.5,col="blue",

panel=function(...){

panel.xyplot(...)

panel.text(x=-10.1,y=2.7,label="SLC6A5",col="blue",cex=0.7,fontface="bold")

panel.text(x=-9.7,y=2.15,label="MYOZ2",col="blue",cex=0.7,fontface="bold")

panel.text(x=-7,y=4.2,label="CLEC3B",col="blue",cex=0.7,fontface="bold")

panel.arrows(x1=-9.03,y1=2.52,x0=-7,y0=4,length=0.05,col="blue")

panel.text(x=-5.8,y=3.15,label="CTSG",col="blue",cex=0.7,fontface="bold")

panel.arrows(x1=-8.65,y1=2.45,x0=-6,y0=3,length=0.05,col="blue")

panel.text(x=-7.45,y=2.1,label="SULT1B1",col="blue",cex=0.7,fontface="bold")

})

width <- 25

height <- 20

tiff(filename = "H:/Volcano_plot.tif",

width = width, height = height, unit="cm", compression = "lzw", bg = "white", res = 800)

print(fig.volcano)

dev.off()

##############################

#### 3. Survival analysis #########

##############################

library(lattice)

library(latticeExtra)

library(xlsx)

library(survival)

countN <- function ( v ) {

length ( v ) - sum ( is.na ( v ) )

}

descriptive_stats <- function (x) {

c(N=countN(x),Mean=mean(x,na.rm=TRUE),SD=sd(x,na.rm=TRUE),Median=median(x,na.rm=TRUE),

IQR=IQR(x,na.rm=TRUE),Min=min(x,na.rm=TRUE),Max=max(x,na.rm=TRUE))}

cex_dot <- 0.5

cex_red <- 0.55

cex_axis <- 1

cex_tick <- 0.8

parstrip <-

list(layout.heights =list(main.key.padding = -2))

parbw <-

list(box.rectangle=list(col="black"),

box.umbrella=list(col="black"),plot.symbol = list(pch=".",col = "white"),

layout.heights =list(main.key.padding = -2,strip = 1.5))

panelbw <- function(...) {

panel.bwplot(...,pch="|")

panel.xyplot(...,jitter.x = TRUE, pch=16, cex=cex_dot, amount=0.15)}

stripwhite <- strip.custom(bg="white")

#### set local drive directories.

setwd("XXXX")

#### diagnosis

data.diag <- read.csv("", header=TRUE, skip=1)

data.diag$Cluster <- factor(data.diag$Cluster)

summary(coxph(Surv(Overall.survival,Event.death)~ Age..years, data=data.diag))

summary(coxph(Surv(Overall.survival,Event.death)~ Neutering.status, data=data.diag))

summary(coxph(Surv(Overall.survival,Event.death)~ Sex, data=data.diag))

summary(coxph(Surv(Overall.survival,Event.death)~ Cluster, data=data.diag))

summary(coxph(Surv(Progression..free.survival ,Event.Progress)~ Age..years, data=data.diag))

summary(coxph(Surv(Progression..free.survival ,Event.Progress)~ Neutering.status, data=data.diag))

summary(coxph(Surv(Progression..free.survival ,Event.Progress)~ Sex, data=data.diag))

summary(coxph(Surv(Progression..free.survival ,Event.Progress)~ Cluster, data=data.diag))

#### treatment

data.trt <- read.csv("", header=TRUE, skip=1)

data.trt$Cluster <- factor(data.trt$Cluster)

summary(coxph(Surv(Overall.survival,Event.death)~ Age..years, data=data.trt))

summary(coxph(Surv(Overall.survival,Event.death)~ Neutering.status, data=data.trt))

summary(coxph(Surv(Overall.survival,Event.death)~ Sex, data=data.trt))

summary(coxph(Surv(Overall.survival,Event.death)~ Cluster, data=data.trt))

summary(coxph(Surv(Progression..free.survival ,Event.Progress)~ Age..years, data=data.trt))

summary(coxph(Surv(Progression..free.survival ,Event.Progress)~ Neutering.status, data=data.trt))

summary(coxph(Surv(Progression..free.survival ,Event.Progress)~ Sex, data=data.trt))

summary(coxph(Surv(Progression..free.survival ,Event.Progress)~ Cluster, data=data.trt))

# between clusters

print(survfit(Surv(Overall.survival,Event.death)~ Cluster, data=data.diag))

summary(coxph(Surv(Overall.survival,Event.death)~ Cluster, data=data.diag))

f1 <- survfit(Surv(Overall.survival,Event.death)~ Cluster, type="kaplan-meier", data=data.diag)

plot(f1, lwd=1,lty=1:2,mark.time=TRUE,cex.axis=0.9,las=1)

title(xlab="Overall survival (days)",ylab="Survival probability",cex.lab=1)

legend(400,1,c("Cluster 1","Cluster 2"),lwd=1,lty=1:2, cex=0.8)

## log rank test

survdiff(Surv(Overall.survival,Event.death)~ Cluster, data=data.diag)

print(survfit(Surv(Progression..free.survival ,Event.Progress)~ Cluster, data=data.diag))

summary(coxph(Surv(Progression..free.survival ,Event.Progress)~ Cluster, data=data.diag))

f2 <- survfit(Surv(Progression..free.survival ,Event.Progress)~ Cluster, type="kaplan-meier", data=data.diag)

plot(f2, lwd=1,lty=1:2,mark.time=TRUE,cex.axis=0.9,las=1)

title(xlab="Progression free survival (days)",ylab="Probability",cex.lab=1)

legend(150,1,c("Cluster 1","Cluster 2"),lwd=1,lty=1:2, cex=0.8)

width <- 10

height <- 10

tiff(filename = "OS.tif",

width = width, height = height, unit="cm", compression = "lzw", bg = "white", res = 500)

f1 <- survfit(Surv(Overall.survival,Event.death)~ Cluster, type="kaplan-meier", data=data1)

plot(f1, lwd=1,lty=1:2,mark.time=TRUE,cex.axis=0.7,las=1)

title(xlab="Overall survival (days)",ylab="Probability",cex.lab=0.8)

legend(400,1,c("Cluster 1","Cluster 2"),lwd=1,lty=1:2, cex=0.7)

dev.off()
