# Supplementary material for: Gene Expression Profiling of B Cell Lymphoma in Dogs Reveals Dichotomous Metabolic Signatures Distinguished by Oxidative Phosphorylation
Source: Front Oncol. 2020 Mar 6;10:307. doi: 10.3389/fonc.2020.00307 (PMC7069556; doi:10.3389/fonc.2020.00307)
Supplement: Supplementary file 5 [file Table_5.DOCX]

**Supplementary Data 5: Sixteen genes overlapped between our dataset and the published canine GCB/ABC-DLBCL gene classifier dataset**

| **Gene Symbol** | **log_2_FC^1^** | **Gene Description** |
| --- | --- | --- |
| FADS2 | 2.94 | fatty acid desaturase 2 |
| PLEKHA8 | 2.65 | Pleckstrin homology domain-containing family A member 8 |
| PGP | 2.54 | phosphoglycolate phosphatase |
| HMGA1 | 2.08 | high mobility group protein HMG-I/HMG-Y |
| TRABD | 1.83 | TraB domain containing |
| MYO9B | 1.61 | myosin IXB |
| TOR1A | -1.37 | torsin family 1 member A |
| NMT1 | -1.4 | N-myristoyltransferase 1 |
| ATP6V0D1 | -1.43 | ATPase H+ transporting V0 subunit d1 |
| MAT2B | -1.43 | methionine adenosyltransferase 2B |
| ATP6AP1 | -1.61 | ATPase H+ transporting accessory protein 1 |
| DERL2 | -1.67 | Derlin 2 |
| ATG4A | -1.73 | autophagy related 4A cysteine peptidase |
| AP3M2 | -1.79 | adaptor related protein complex 3 mu 2 subunit |
| GSTM4 | -1.86 | glutathione S-transferase mu 4 |
| NFATC2 | -1.9 | nuclear factor of activated T-cells 2 |

^1^fold change values of canine RNA sequencing data of this study
